# *N*,*N*′-Dineopentyl­naphthalene-1,8-diamine

**DOI:** 10.1107/S1600536809050867

**Published:** 2009-12-04

**Authors:** Ilia A. Guzei, Lara C. Spencer, Nicholas J. Hill

**Affiliations:** a Department of Chemistry, University of Wisconsin-Madison, 1101 University Ave, Madison, WI 53706, USA

## Abstract

In the title compound, C_20_H_30_N_2_, all bond distances and angles fall within the usual ranges but the C(*ipso*)—N distances [1.391 (5) and 1.398 (4) Å] are slightly shorter than the corresponding typical average distance of 1.42 (3) Å. The N atoms may be described as pyramidal *sp* ^3^-hybridized with an N—H⋯H—N separation of 2.07 (2) Å. This is necessitated because the two C(bridgehead)—C(*ipso*)—N—C torsion angles [170.6 (4) and 172.6 (3)°] would require the amine H atoms to be in prohibitively close proximity if the N atoms were assumed to be *sp* ^2^-hybridized.

## Related literature

For the use of 1,8-bis­(diamido)naphthalene (DAN) ligands in the preparation of thermally stable *N*-heterocyclic carbenes, germylenes and stannylenes, see: Avent *et al.* (2004 ▶); Bazinet *et al.* (2001*a* ▶,*b* ▶, 2003 ▶, 2007 ▶). For our studies on *N*-heterocyclic silylenes, see: Hill *et al.* (2005 ▶); Li *et al.* (2006 ▶); Naka *et al.* (2004 ▶). For DAN ligands in transition metal coordination chemistry, see: Lavoie *et al.* (2007 ▶); Bazinet *et al.* (2001*b* ▶). Their titanium and zirconium complexes have been found to be effective catalysts for olefin polymerization, see: Lee *et al.* (2001 ▶). For a description of the Cambridge Structural Database, see: Allen (2002 ▶). For geometrical analysis, see: Bruno *et al.* (2002 ▶). For the preparation of the title compound, see: Daniele *et al.* (2001 ▶).
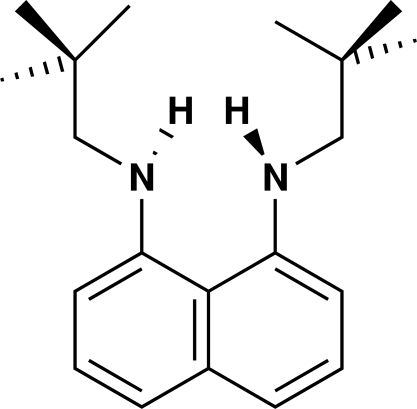


## Experimental

### 

#### Crystal data

C_20_H_30_N_2_
*M* *_r_* = 298.46
Orthorhombic, 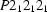

*a* = 6.0425 (14) Å
*b* = 16.196 (3) Å
*c* = 18.861 (4) Å
*V* = 1845.8 (7) Å^3^
*Z* = 4
Mo *K*α radiation
μ = 0.06 mm^−1^
*T* = 300 K
0.70 × 0.30 × 0.20 mm

#### Data collection

Bruker SMART X2S diffractometer
Absorption correction: multi-scan (*SADABS*; Bruker, 2009 ▶) *T*_min_ = 0.958, *T*_max_ = 0.988
12752 measured reflections
2042 independent reflections
1203 reflections with *I* > 2σ(*I*)
*R*_int_ = 0.090

#### Refinement

*R*[*F* ^2^ > 2σ(*F* ^2^)] = 0.053
*wR*(*F* ^2^) = 0.140
*S* = 0.93
2042 reflections
213 parameters
6 restraints
H atoms treated by a mixture of independent and constrained refinement
Δρ_max_ = 0.12 e Å^−3^
Δρ_min_ = −0.13 e Å^−3^

### 

Data collection: *GIS* (Bruker, 2009 ▶); cell refinement: *SAINT* (Bruker, 2009 ▶); data reduction: *SAINT*; program(s) used to solve structure: *SHELXTL* (Sheldrick, 2008 ▶); program(s) used to refine structure: *SHELXTL* and *OLEX2* (Dolomanov *et al.*, 2009 ▶); molecular graphics: *SHELXTL*; software used to prepare material for publication: *SHELXTL*, *publCIF* (Westrip, 2009 ▶) and *modiCIFer* (Guzei, 2007 ▶).

## Supplementary Material

Crystal structure: contains datablocks global, I. DOI: 10.1107/S1600536809050867/zs2021sup1.cif

Structure factors: contains datablocks I. DOI: 10.1107/S1600536809050867/zs2021Isup2.hkl

Additional supplementary materials:  crystallographic information; 3D view; checkCIF report

## Figures and Tables

**Fig. 1.**
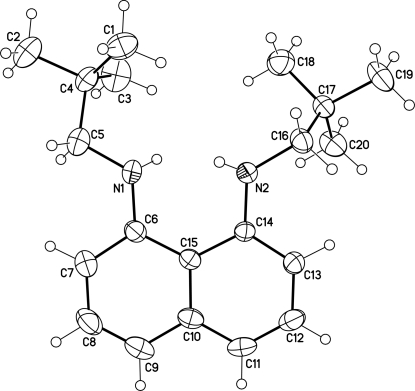
The molecular structure of (I) with the thermal ellipsoids shown at 30% probability level.

## supplementary crystallographic information

### Comment

1,8-Bis(diamido)naphthalene (DAN) ligands have recently been employed in the preparation of thermally stable N-heterocyclic carbenes, germylenes, and stannylenes (Avent *et al.*, 2004; Bazinet *et al.*, 2007; Bazinet *et al.*, 2003; Bazinet *et al.*, 2001*a*). As a continuation of our study of N-heterocyclic silylenes (Hill *et al.*, 2005; Li *et al.*, 2006; Naka *et al.*, 2004), we sought to prepare the corresponding silicon compound. The title compound, C_20_H_30_N_2_ (I), was selected as a scaffold on account of the potentially greater steric protection afforded the reactive silicon(II) atom by the neopentyl group compared to the more common isopropyl analog. Our results in this area will be presented elsewhere.

DAN ligands have also been examined in the realm of transition metal coordination chemistry (*e.g.* Lavoie *et al.*, 2007, Bazinet *et al*., 2001*b*). Their titanium and zirconium complexes have been found to be effective catalysts for olefin polymerization (Lee *et al.*, 2001).

In the structure of the title compound (Fig. 1), all bond distances and angles fall in the usual ranges (Bruno *et al.*, 2002). The C(*ipso*)-N distances [1.391 (5) and 1.398 (4) Å] are slightly shorter than the corresponding distance of 1.42 (3) Å, obtained by averaging 228 distances in 62 related compounds reported to the Cambridge Structural Database (CSD) (Allen, 2002). However, the difference is not statistically significant. An important feature of the structure is the location of the amine H atoms. Their positions are dependent on intermolecular hydrogen bonding (absent in our case), torsion angle φ [ = C(bridgehead)-C(*ipso*)-N—C], and hybridization of the N atoms. The φ angles in (I) [170.6 (4) and 172.6 (3)°], would require the amine H atoms to be in prohibitively close proximity if the N atoms are assumed to be *sp*^2^-hybridized. To avoid the steric conflict the N atoms are described as pyramidal *sp*^3^-hybridized with the N1—H1···H2—N2 separation of 2.07 (2) Å. In several related structures of DAN-derivatives reported to the CSD this problem is absent as the relative location of the N-substituents allows for intramolecular N—H···N hydrogen bonding. This is illustrated with dissimilar pairs of the φ angles: 84.5 and -114.3° in HEYHEY, 92.0 and 107.8° in NAKKAL, 107.0 and 95.6° in NAKKEP, 92.9 and -178.1° in NUPJEN, 67.9 and 167.2° in OHOKAX, -119.1 and 158.0° in KOCTEC.

### Experimental

The title compound was obtained by treatment of 1,8-diaminonaphthalene with 2,2-dimethylpropanoyl chloride followed by LiAlH_4_ reduction and aqueous work-up according to the procedure of Daniele *et al.* (2001). ^1^H– and ^13^C{^1^H}-NMR data were in agreement with literature values. Needle-like crystals suitable for X-ray diffraction studies were obtained from a solution of the title compound stored at -20 °C in THF for several weeks.

### Refinement

All non-amine H-atoms were placed in idealized locations and refined as riding with appropriate thermal displacement coefficients *U*_iso_(H) = 1.2 or 1.5 times *U*_eq_(bearing atom). The amine H atoms were refined with the N—H distances restrained to 0.880 (1) Å and unconstrained thermal displacement coefficient. The refinement was performed with an 'anti-bumping' restraint. The Friedel pairs were merged.

### Crystal data

**Table:**

C_20_H_30_N_2_	*F*(000) = 656
*M**_r_* = 298.46	*D*_x_ = 1.074 Mg m^−^^3^
Orthorhombic, *P*2_1_2_1_2_1_	Mo *K*α radiation, λ = 0.71073 Å
Hall symbol: P 2ac 2ab	Cell parameters from 1989 reflections
*a* = 6.0425 (14) Å	θ = 2.5–20.6°
*b* = 16.196 (3) Å	µ = 0.06 mm^−^^1^
*c* = 18.861 (4) Å	*T* = 300 K
*V* = 1845.8 (7) Å^3^	Needle, yellow
*Z* = 4	0.70 × 0.30 × 0.20 mm

### Data collection

**Table:**

Bruker SMART X2S diffractometer	2042 independent reflections
Radiation source: micro-focus sealed tube	1203 reflections with *I* > 2σ(*I*)
doubly curved silicon crystal	*R*_int_ = 0.090
ω scans	θ_max_ = 25.7°, θ_min_ = 2.5°
Absorption correction: multi-scan (*SADABS*; Bruker, 2009)	*h* = −7→7
*T*_min_ = 0.958, *T*_max_ = 0.988	*k* = −19→15
12752 measured reflections	*l* = −22→22

### Refinement

**Table:**

Refinement on *F*^2^	Primary atom site location: structure-invariant direct methods
Least-squares matrix: full	Secondary atom site location: difference Fourier map
*R*[*F*^2^ > 2σ(*F*^2^)] = 0.053	Hydrogen site location: inferred from neighbouring sites
*wR*(*F*^2^) = 0.140	H atoms treated by a mixture of independent and constrained refinement
*S* = 0.93	*w* = 1/[σ^2^(*F*_o_^2^) + (0.079*P*)^2^] where *P* = (*F*_o_^2^ + 2*F*_c_^2^)/3
2042 reflections	(Δ/σ)_max_ < 0.001
213 parameters	Δρ_max_ = 0.12 e Å^−^^3^
6 restraints	Δρ_min_ = −0.13 e Å^−^^3^

### Special details

**Table:**

Geometry. All e.s.d.'s (except the e.s.d. in the dihedral angle between two l.s. planes) are estimated using the full covariance matrix. The cell e.s.d.'s are taken into account individually in the estimation of e.s.d.'s in distances, angles and torsion angles; correlations between e.s.d.'s in cell parameters are only used when they are defined by crystal symmetry. An approximate (isotropic) treatment of cell e.s.d.'s is used for estimating e.s.d.'s involving l.s. planes.
Refinement. Refinement of *F*^2^ against ALL reflections. The weighted *R*-factor *wR* and goodness of fit *S* are based on *F*^2^, conventional *R*-factors *R* are based on *F*, with *F* set to zero for negative *F*^2^. The threshold expression of *F*^2^ > σ(*F*^2^) is used only for calculating *R*-factors(gt) *etc*. and is not relevant to the choice of reflections for refinement. *R*-factors based on *F*^2^ are statistically about twice as large as those based on *F*, and *R*- factors based on ALL data will be even larger.

### Fractional atomic coordinates and isotropic or equivalent isotropic displacement parameters (Å^2^)

**Table:**

	*x*	*y*	*z*	*U*_iso_*/*U*_eq_	
N1	0.3691 (7)	0.6434 (2)	0.30061 (17)	0.0693 (11)	
H1	0.309 (10)	0.5953 (17)	0.299 (2)	0.16 (3)*	
N2	0.2799 (6)	0.55146 (19)	0.41609 (17)	0.0513 (8)	
H2	0.396 (2)	0.578 (2)	0.404 (2)	0.13 (2)*	
C1	0.7600 (7)	0.5450 (3)	0.2652 (2)	0.0726 (12)	
H1C	0.8605	0.5866	0.2818	0.109*	
H1A	0.8424	0.4997	0.2456	0.109*	
H1B	0.6714	0.5256	0.3040	0.109*	
C2	0.7503 (10)	0.6090 (3)	0.1446 (2)	0.1015 (18)	
H2C	0.8566	0.6493	0.1599	0.152*	
H2B	0.6562	0.6328	0.1091	0.152*	
H2A	0.8262	0.5621	0.1252	0.152*	
C3	0.4470 (9)	0.5158 (3)	0.1828 (2)	0.0908 (16)	
H3A	0.5274	0.4699	0.1633	0.136*	
H3C	0.3521	0.5387	0.1471	0.136*	
H3B	0.3592	0.4972	0.2221	0.136*	
C4	0.6097 (7)	0.5816 (2)	0.20806 (19)	0.0543 (10)	
C5	0.4883 (9)	0.6577 (3)	0.2348 (2)	0.0725 (13)	
H5B	0.5946	0.7018	0.2421	0.087*	
H5A	0.3844	0.6758	0.1988	0.087*	
C6	0.2063 (6)	0.6975 (2)	0.32437 (18)	0.0467 (10)	
C7	0.1708 (8)	0.7721 (2)	0.2898 (2)	0.0649 (12)	
H7	0.2658	0.7881	0.2535	0.078*	
C8	−0.0050 (9)	0.8235 (3)	0.3087 (2)	0.0762 (14)	
H8	−0.0252	0.8730	0.2846	0.091*	
C9	−0.1462 (8)	0.8027 (2)	0.3611 (2)	0.0677 (12)	
H9	−0.2647	0.8371	0.3719	0.081*	
C10	−0.1146 (6)	0.7286 (2)	0.39979 (19)	0.0484 (10)	
C11	−0.2626 (7)	0.7085 (3)	0.4547 (2)	0.0574 (11)	
H11	−0.3817	0.7432	0.4643	0.069*	
C12	−0.2334 (7)	0.6394 (2)	0.4938 (2)	0.0594 (11)	
H12	−0.3333	0.6264	0.5296	0.071*	
C13	−0.0538 (6)	0.5874 (2)	0.48056 (19)	0.0507 (10)	
H13	−0.0362	0.5403	0.5083	0.061*	
C14	0.0986 (6)	0.60337 (19)	0.42771 (17)	0.0389 (8)	
C15	0.0688 (6)	0.67558 (19)	0.38365 (17)	0.0394 (8)	
C16	0.3391 (6)	0.4827 (2)	0.46264 (18)	0.0519 (10)	
H16B	0.2712	0.4920	0.5086	0.062*	
H16A	0.4982	0.4833	0.4695	0.062*	
C17	0.2717 (6)	0.3962 (2)	0.43668 (18)	0.0463 (9)	
C18	0.3875 (8)	0.3775 (3)	0.3663 (2)	0.0758 (13)	
H18B	0.3323	0.4139	0.3303	0.114*	
H18A	0.3588	0.3213	0.3529	0.114*	
H18C	0.5440	0.3855	0.3716	0.114*	
C19	0.3484 (8)	0.3340 (2)	0.4927 (2)	0.0747 (14)	
H19A	0.3080	0.2793	0.4781	0.112*	
H19C	0.2791	0.3464	0.5372	0.112*	
H19B	0.5062	0.3373	0.4978	0.112*	
C20	0.0236 (7)	0.3885 (2)	0.4256 (2)	0.0684 (12)	
H20C	−0.0510	0.3955	0.4701	0.103*	
H20A	−0.0099	0.3350	0.4066	0.103*	
H20B	−0.0252	0.4303	0.3930	0.103*	

### Atomic displacement parameters (Å^2^)

**Table:**

	*U*^11^	*U*^22^	*U*^33^	*U*^12^	*U*^13^	*U*^23^
N1	0.082 (3)	0.059 (2)	0.067 (2)	0.013 (2)	0.035 (2)	0.0214 (17)
N2	0.049 (2)	0.0449 (19)	0.060 (2)	0.0077 (17)	0.0144 (18)	0.0112 (15)
C1	0.057 (3)	0.087 (3)	0.074 (3)	−0.001 (3)	−0.001 (3)	−0.017 (2)
C2	0.102 (4)	0.118 (4)	0.084 (3)	0.004 (4)	0.043 (4)	0.008 (3)
C3	0.093 (4)	0.105 (4)	0.074 (3)	−0.028 (3)	−0.018 (3)	−0.010 (3)
C4	0.053 (2)	0.064 (3)	0.046 (2)	−0.007 (2)	0.009 (2)	−0.0063 (18)
C5	0.081 (3)	0.074 (3)	0.062 (3)	−0.001 (3)	0.020 (3)	0.011 (2)
C6	0.051 (2)	0.041 (2)	0.048 (2)	0.0028 (19)	−0.0014 (19)	0.0013 (17)
C7	0.076 (3)	0.053 (3)	0.066 (3)	−0.001 (2)	0.006 (2)	0.012 (2)
C8	0.098 (4)	0.052 (3)	0.079 (3)	0.018 (3)	−0.006 (3)	0.016 (2)
C9	0.073 (3)	0.049 (3)	0.081 (3)	0.018 (2)	−0.014 (3)	−0.005 (2)
C10	0.048 (2)	0.041 (2)	0.056 (2)	0.0026 (19)	−0.011 (2)	−0.0122 (17)
C11	0.042 (2)	0.061 (3)	0.070 (3)	0.009 (2)	0.004 (2)	−0.021 (2)
C12	0.055 (3)	0.056 (3)	0.067 (3)	−0.006 (2)	0.020 (2)	−0.017 (2)
C13	0.062 (3)	0.041 (2)	0.049 (2)	−0.001 (2)	0.009 (2)	0.0002 (17)
C14	0.046 (2)	0.0346 (19)	0.0361 (18)	−0.0018 (17)	0.0020 (18)	−0.0066 (15)
C15	0.043 (2)	0.0343 (18)	0.0413 (19)	−0.0026 (17)	−0.0055 (18)	−0.0056 (15)
C16	0.048 (2)	0.047 (2)	0.061 (2)	−0.0022 (19)	−0.001 (2)	0.0096 (18)
C17	0.043 (2)	0.043 (2)	0.053 (2)	−0.0013 (18)	−0.0042 (19)	0.0048 (17)
C18	0.079 (3)	0.065 (3)	0.083 (3)	0.000 (3)	0.013 (3)	−0.005 (2)
C19	0.071 (3)	0.056 (3)	0.097 (3)	0.002 (2)	−0.019 (3)	0.026 (2)
C20	0.054 (3)	0.060 (3)	0.091 (3)	−0.004 (2)	−0.011 (2)	0.004 (2)

### Geometric parameters (Å, °)

**Table:**

N1—C6	1.391 (5)	C9—C10	1.418 (5)
N1—C5	1.453 (5)	C9—H9	0.9300
N1—H1	0.86 (3)	C10—C11	1.407 (5)
N2—C14	1.398 (4)	C10—C15	1.434 (5)
N2—C16	1.463 (4)	C11—C12	1.352 (5)
N2—H2	0.86 (3)	C11—H11	0.9300
C1—C4	1.528 (5)	C12—C13	1.396 (5)
C1—H1C	0.9600	C12—H12	0.9300
C1—H1A	0.9600	C13—C14	1.382 (5)
C1—H1B	0.9600	C13—H13	0.9300
C2—C4	1.533 (5)	C14—C15	1.446 (4)
C2—H2C	0.9600	C16—C17	1.537 (5)
C2—H2B	0.9600	C16—H16B	0.9700
C2—H2A	0.9600	C16—H16A	0.9700
C3—C4	1.526 (6)	C17—C20	1.519 (5)
C3—H3A	0.9600	C17—C18	1.531 (5)
C3—H3C	0.9600	C17—C19	1.532 (5)
C3—H3B	0.9600	C18—H18B	0.9600
C4—C5	1.521 (6)	C18—H18A	0.9600
C5—H5B	0.9700	C18—H18C	0.9600
C5—H5A	0.9700	C19—H19A	0.9600
C6—C7	1.390 (5)	C19—H19C	0.9600
C6—C15	1.438 (5)	C19—H19B	0.9600
C7—C8	1.396 (6)	C20—H20C	0.9600
C7—H7	0.9300	C20—H20A	0.9600
C8—C9	1.349 (6)	C20—H20B	0.9600
C8—H8	0.9300		
			
C6—N1—C5	121.7 (3)	C11—C10—C9	119.3 (4)
C6—N1—H1	107 (5)	C11—C10—C15	120.6 (3)
C5—N1—H1	108 (4)	C9—C10—C15	120.1 (4)
C14—N2—C16	123.7 (3)	C12—C11—C10	120.6 (4)
C14—N2—H2	112 (3)	C12—C11—H11	119.7
C16—N2—H2	110.6 (14)	C10—C11—H11	119.7
C4—C1—H1C	109.5	C11—C12—C13	120.3 (4)
C4—C1—H1A	109.5	C11—C12—H12	119.9
H1C—C1—H1A	109.5	C13—C12—H12	119.9
C4—C1—H1B	109.5	C14—C13—C12	122.3 (3)
H1C—C1—H1B	109.5	C14—C13—H13	118.9
H1A—C1—H1B	109.5	C12—C13—H13	118.9
C4—C2—H2C	109.5	C13—C14—N2	121.5 (3)
C4—C2—H2B	109.5	C13—C14—C15	118.9 (3)
H2C—C2—H2B	109.5	N2—C14—C15	119.6 (3)
C4—C2—H2A	109.5	C10—C15—C6	117.6 (3)
H2C—C2—H2A	109.5	C10—C15—C14	117.3 (3)
H2B—C2—H2A	109.5	C6—C15—C14	125.1 (3)
C4—C3—H3A	109.5	N2—C16—C17	115.9 (3)
C4—C3—H3C	109.5	N2—C16—H16B	108.3
H3A—C3—H3C	109.5	C17—C16—H16B	108.3
C4—C3—H3B	109.5	N2—C16—H16A	108.3
H3A—C3—H3B	109.5	C17—C16—H16A	108.3
H3C—C3—H3B	109.5	H16B—C16—H16A	107.4
C5—C4—C3	111.0 (4)	C20—C17—C18	108.4 (4)
C5—C4—C1	111.5 (3)	C20—C17—C19	109.9 (3)
C3—C4—C1	109.4 (4)	C18—C17—C19	109.2 (3)
C5—C4—C2	107.0 (3)	C20—C17—C16	112.3 (3)
C3—C4—C2	108.4 (3)	C18—C17—C16	109.6 (3)
C1—C4—C2	109.5 (4)	C19—C17—C16	107.4 (3)
N1—C5—C4	113.2 (3)	C17—C18—H18B	109.5
N1—C5—H5B	108.9	C17—C18—H18A	109.5
C4—C5—H5B	108.9	H18B—C18—H18A	109.5
N1—C5—H5A	108.9	C17—C18—H18C	109.5
C4—C5—H5A	108.9	H18B—C18—H18C	109.5
H5B—C5—H5A	107.8	H18A—C18—H18C	109.5
C7—C6—N1	120.3 (4)	C17—C19—H19A	109.5
C7—C6—C15	119.4 (4)	C17—C19—H19C	109.5
N1—C6—C15	120.3 (3)	H19A—C19—H19C	109.5
C6—C7—C8	121.0 (4)	C17—C19—H19B	109.5
C6—C7—H7	119.5	H19A—C19—H19B	109.5
C8—C7—H7	119.5	H19C—C19—H19B	109.5
C9—C8—C7	121.4 (4)	C17—C20—H20C	109.5
C9—C8—H8	119.3	C17—C20—H20A	109.5
C7—C8—H8	119.3	H20C—C20—H20A	109.5
C8—C9—C10	120.2 (4)	C17—C20—H20B	109.5
C8—C9—H9	119.9	H20C—C20—H20B	109.5
C10—C9—H9	119.9	H20A—C20—H20B	109.5
			
C6—N1—C5—C4	−163.9 (4)	C16—N2—C14—C13	−7.4 (5)
C3—C4—C5—N1	69.2 (5)	C16—N2—C14—C15	172.6 (3)
C1—C4—C5—N1	−53.1 (5)	C11—C10—C15—C6	176.7 (3)
C2—C4—C5—N1	−172.7 (4)	C9—C10—C15—C6	−4.4 (5)
C5—N1—C6—C7	−7.5 (6)	C11—C10—C15—C14	−2.3 (5)
C5—N1—C6—C15	170.6 (4)	C9—C10—C15—C14	176.6 (3)
N1—C6—C7—C8	173.9 (4)	C7—C6—C15—C10	6.2 (5)
C15—C6—C7—C8	−4.2 (6)	N1—C6—C15—C10	−171.9 (3)
C6—C7—C8—C9	0.1 (7)	C7—C6—C15—C14	−174.8 (3)
C7—C8—C9—C10	1.8 (7)	N1—C6—C15—C14	7.0 (5)
C8—C9—C10—C11	179.4 (4)	C13—C14—C15—C10	2.6 (4)
C8—C9—C10—C15	0.4 (6)	N2—C14—C15—C10	−177.4 (3)
C9—C10—C11—C12	−178.2 (4)	C13—C14—C15—C6	−176.4 (3)
C15—C10—C11—C12	0.7 (5)	N2—C14—C15—C6	3.7 (5)
C10—C11—C12—C13	0.8 (6)	C14—N2—C16—C17	101.3 (4)
C11—C12—C13—C14	−0.5 (6)	N2—C16—C17—C20	−59.1 (4)
C12—C13—C14—N2	178.7 (3)	N2—C16—C17—C18	61.5 (4)
C12—C13—C14—C15	−1.2 (5)	N2—C16—C17—C19	−180.0 (3)
